# The Safety of Herbal Medicine: From Prejudice to Evidence

**DOI:** 10.1155/2015/316706

**Published:** 2015-03-09

**Authors:** Junhua Zhang, Igho J. Onakpoya, Paul Posadzki, Mohamed Eddouks

**Affiliations:** ^1^Tianjin University of Traditional Chinese Medicine, Tianjin 300193, China; ^2^Department of Primary Care Health Sciences, University of Oxford, Oxford OX2 6GG, UK; ^3^Lee Kong Chian School of Medicine, Singapore 138543; ^4^Lee Kong Chian School of Medicine, Singapore 138543 Moulay Ismail University, 52000 Errachidia, Morocco

About 100 years ago, natural herbs were the main remedy for treating human diseases. It has been estimated that 25% of modern medicines are made from plants first used traditionally [1], such as aspirin, artemisinin, ephedrine, and paclitaxel. However, there is limited scientific evidence to establish the safety and efficacy of most herbal products [2]. With the wide application of chemical drugs, herbal medicine and other traditional therapies have shown sharp contraction. As a country with rich herbal resource, China is not an exception. In recent decades, spectrum of disease has shifted and the complex chronic diseases have become the main part. The effect of Western medicine treatment is not satisfactory and problems of the adverse drug reaction are also very prominent. The complementary and alternative treatment, especially the herbal medicine, has gained more attention and has also become popular.

About 80% of people worldwide rely on herbal medicines for some aspects of their primary health care [1]. In 2008, the global market for herbal remedies was about USD 83 billion, and now it is about 100 billion (see http://www.nutraceuticalsworld.com/). In China, the industry output value of Chinese patent medicines reached about USD 80 billion in 2013.

*(1) Safety Issues of Herbal Medicine*. Along with the significant increase of worldwide consumption, the safety of herbal medicine has been highlighted. At present, there are misunderstanding and prejudice toward the safety of herbal medicine. So, objective understanding, neutral and fair interpretation, and publicity are warranted. 

*(a) Herbal Medicine Is Drug, Not Food*. Advocates will advertise that herbal medicine originated from nature and belongs to green therapy and has no toxin or adverse effect and people can take it in the long term and so forth. These sayings are slogans of the advocates which have misled people with less medical knowledge. On one hand, it will lead to many severe adverse events by misusing herbal medicine; on the other hand, it will cause people's panic and anxiety due to some adverse events reports. We should clearly recognize that herbal medicinal products are widely considered to be of lower risk compared with synthetic drugs; they are not completely free from the possibility of toxicity or adverse effects. Exaggerated propaganda and giving up using for adverse event are prejudice against herbal medicine. Therefore, to ensure the safety use of herbal medicinal products, herbal medicine should be managed as drug. 

*(b) The Relative Property of Herbal Medicine Safety.* As the Chinese saying goes, “all medicines have their own side effects”; that is, medicine is a double-blade sword: it can cure disease or maintain health, while it may also cause damage to human body. All effective drugs may produce adverse drug reactions; herbal medicines are no exception. Herbal medicine should be adopted by appropriate dosage and course of treatment and for adapted syndrome, rather than unrestricted abusing. Overdosage and course of treatment are bound to safety problems. The toxicity of* Radix Bupleuri Chinensis* in Japan has attracted worldwide attentions. A research on the quantity-toxin relation indicates that the toxic dose of* Radix Bupleuri Chinensis* (192 g/60 kg) is much greater than clinical common dose (9 g/60 kg). However, high-dose and long-term use may also cause adverse event [3]. 

*(c) The Complexity of Safety of Herbal Medicine*. There are a number of causes of adverse events to herbal medicines, which can be divided into “direct” and “indirect” reasons.

*Intrinsic Toxicity*. Direct reason is the intrinsic toxicity of some herb at normal therapeutic dosage or in overdose. Adverse reactions associated with Ephedra,* Aristolochia*, and* Aconitum* have shown that herbs can produce toxicity in humans.

*External Toxicity*. Adverse effects associated with herbal medicines may result from contamination of products with toxic metals, adulteration, misidentification or substitution of herbal ingredients, or improperly processed or prepared products [4]. For example,* Caulis Akebiae* replaced by* Caulis Aristolochiae Manshuriensis* and* Stephania tetrandra* replaced by* Aristolochia fangchi* have led to the serious problem of “aristolochic acid nephropathy.”

*Wrong Indication*. Inappropriate use of herbal medicines can cause negative or dangerous effects. For instance, the herb “Ma Huang” (Ephedra) is traditionally used in China to treat respiratory congestion, while it was marketed as dietary supplements formulated for weight reduction in US. Overdosage use led to at least a dozen deaths, heart attacks, and strokes [5, 6]. 

*Herb-Drug Interaction*. All herbal medicines are complex mixtures of more than one active ingredient. Multitude of active ingredients will increase the possibilities of interactions between herbal medicines and conventional drugs. Moreover, users of medicinal herbs are usually suffering from chronic conditions for which they are likely to take prescribed drugs concomitantly. This, in turn, further increases the potential of herb-drug interaction [7]. A retrospective cross-sectional study found that the prevalence of concomitant herbal medicinal products and antipsychotic treatment was 36.4% (34.2%–38.6%). Herbal medicine regimens containing* Radix Bupleuri, Fructus Gardenia, Fructus Schisandrae Chinensis, Radix Rehmanniae, Akebia Caulis, and Semen Plantaginis* in concomitant use with quetiapine, clozapine, and olanzapine were associated with nearly 60% of the risk of adverse outcomes [8]. 

*(d) Weak Basic Research in Safety of Herbal Medicine*.* Sheng Nong's Herbal, *the first classic of Chinese materia medica, recorded 365 herbs, which were divided into three levels (high grade, moderate, and inferior) according to the toxin size of each herb. In the Chinese Pharmacopoeia 2010, there are 83 types of Chinese materia medica officially recorded and defined as toxic and they were classified into three categories: high toxicity, medium toxicity, and low toxicity. Efficacy and toxicity of the majority of them are mostly based on traditional knowledge and clinical experience. The toxicity classification is lack of scientific standard and objective experimental data. There is no adequate data about toxic herbs, toxic target organs, safe dose range, safety window of effective dose, and minimum toxic dose. Thus, to specify the toxic and adverse effect of each herbal medicine is a vital base to ensure the safe use of herbal medicine.

Processing of herbal slices is an important step to decrease toxicity. For example, heating processing can make the bitter almond enzyme lose activity and then decrease the toxicity of almond which contains cyanophoric glycoside. Diester-diterpenoid* Aconitum* Alkaloids are the strongest toxicity constituents of aconite and monkshood. By heating processing, diester-diterpenoid* Aconitum* Alkaloids can be decomposed into low toxicity monoester-diterpenoid* Alkaloids* and aconine which are of low toxicity and almost not toxic. If preparation was neglected, risk of aconite will increase. Compatibility, which aims to decrease toxicity and improve treating effect for prescription, is the key theory of Chinese herbal medicine. For instance, combining Radix aconiti praeparata with licorice,* Aconitum* Alkaloids can decrease obviously [9]. Ginger can antagonize the toxicity of* Rhizoma Pinelliae* [10]. Therefore, the scientific connotation of compatibility and preparation of herbal medicine should be investigated deeply. 

*(2) Content of the Special Issue*. This special issue wished to recruit high level research articles, including systematic reviews of herbal medicine safety, case observation and monitoring of adverse events, herbal toxic composition, relations of amount and toxicity, the metabolic process, herb-drug interactions, and methods for risk control. However, we have only received 23 articles for this issue and 12 were rejected due to the content being not closely related with our topic issue or low quality. Finally, 11 articles have been published after being reviewed and revised by the editorial committee and reviewers. Although there are a limited number of papers, the contents are extensive, covering the safety evaluation method, herb-drug interaction, safety evaluation of skin-applied herbal medicine, and the influence of herbal ingredients to cytochrome P450 system, which have certain representations for the related work in this field. 

*(3) Perspective.* Based on the current situation, worldwide research on herbal medicine safety is still not broad or deep enough. For next step, more attention should be paid to researches on the toxicity and the herb-drug interaction of commonly used herb medicines, which are the most necessary and urgent work.

For clinical safety monitoring, spontaneous reporting system or active pharmacovigilance is effective in identifying therapeutically relevant safety issues. Even in countries where herbal medicinal products are regularly assessed before market authorisation, pharmacovigilance is a critical activity to promote the safe use of herbal medicines throughout their life cycle.

A regulatory framework for herbal medicines can provide greater assurance to consumers. However, the regulation and specification of herbal medicines vary significantly different countries. Herbal medicines were managed as food supplement, functional food, health products, or drugs, which caused differential standards and chaotic market. In order to ensure the quality and safety of herbal medicines, the World Health Organization should propose global unified planning, which includes global management standards and quality standards, radical source of herbs, seed and seedling breeding, planting, harvesting and storage, rational proceeding, manufacture, and quality standards. Moreover, safety guarantee system comprised rational clinical practice and risk monitoring should be established to improve the safety of herbal medicine and to play more important role in maintaining human health.

*Junhua Zhang* *Junhua Zhang* *Igho J. Onakpoya* *Igho J. Onakpoya* *Paul Posadzki* *Paul Posadzki* *Mohamed Eddouks* *Mohamed Eddouks*
